# PHYSICAL ACTIVITY IN DEMENTIA PATIENTS AND CAREGIVERS

**DOI:** 10.1093/geroni/igac059.2884

**Published:** 2022-12-20

**Authors:** Prabhash Kakarla, Madieska Ramirez, Jessica Alvarez, Marzena Gieniusz, Ann Swartz, Sergey Tarima, Chris Nouryan, Edith Burns

**Affiliations:** Hofstra University, New Hyde Park, New York, United States; Hofstra-Northwell, New Hyde Park, New York, United States; Hofstra-Northwell, New Hyde Park, New York, United States; Hofstra-Northwell, New Hyde Park, New York, United States; University of Wisconsin-Milwaukee, Milwaukee, Wisconsin, United States; PhD., Milwaukee, Wisconsin, United States; Zucker School of Medicine at Hofstra Northwell, Uniondale, New York, United States; Zucker School of Medicine at Hofstra Northwell, Manhasset, New York, United States

## Abstract

Many studies have explored the benefits of physical activity (PA) in mitigating risk for and symptoms of dementia. However, there are challenges to implementing PA with dementia patients. We conducted an acceptability/feasibility study of PA in patients with Dementia and their caregivers. A semi-structured telephone interview was conducted. Questions piloted with 2 independent faculty for content validity. A random sample of 10 participants was recruited from patient-caregiver dyads enrolled in an Alzheimer’s Dementia Care program (ADCP) at an academic geriatrics practice. Responses manually recorded and personal health information omitted. Descriptive statistics generated using SPSS and response themes categorized by the authors. Major themes included lack of time for caregivers; time requirement would increase caregiver burden (50%). Caregivers of patients with home health aides were less likely to consider time a barrier. Other themes included functional status as a challenge (40%), difficulty motivating patient (40%), unsteady gait (20%), difficulty using devices for video-based exercise (20%). Caregivers expressed preference for asynchronous, in-home PA that could be adapted to their schedules and patient capabilities. Although PA is beneficial for patients with dementia, multiple challenges to PA implementation exist. Main barriers are time requirements and greater caregiver burden. Time would be required to motivate patients, assist with managing technology, and functional impairment that poses potential risk of injury (e.g., gait instability). Engaging Home Health Aides in PA programs could address some of these barriers. Alternative PA (i.e.: chair exercises) could be a solution. Responses will be utilized to modify a future PA intervention.

